# African Trypanosomiasis Gambiense, Italy

**DOI:** 10.3201/eid1111.050649

**Published:** 2005-11

**Authors:** Zeno Bisoffi, Anna Beltrame, Geraldo Monteiro, Alessandra Arzese, Stefania Marocco, Giada Rorato, Mariella Anselmi, Pierluigi Viale

**Affiliations:** * Centre for Tropical Diseases at Sacro Cuore Hospital of Negrar, Verona, Italy; † Clinic of Infectious Diseases at University Hospital, Udine, Italy; ‡ University of Udine Medical School, Udine, Italy

**Keywords:** African trypanosomiasis, *Trypanosoma brucei gambiense*, Italy, dispatch

## Abstract

African trypanosomiasis caused by *Trypanosoma brucei gambiense* has not been reported in Italy. We report 2 cases diagnosed in the summer of 2004. Theses cases suggest an increased risk for expatriates working in trypanosomiasis-endemic countries. Travel medicine clinics should be increasingly aware of this potentially fatal disease.

Human African trypanosomiasis (HAT), also known as sleeping sickness, is caused by a flagellated trypanosome protozoan and transmitted by *Glossina* (tsetse) flies. It is classified into 3 subspecies: *Trypanosoma brucei gambiense*, *T. brucei rhodesiense*, and *T. brucei brucei* (the third subspecies is not pathogenic to humans). These subspecies cannot be distinguished morphologically. *T. b. gambiense*, which is found in western and central Africa, causes chronic disease, while *T. b. rhodesiense*, which is found in eastern and southern Africa, causes acute severe disease. The epidemiology of these subspecies also differs and follows distribution of their main vectors, *Glossina palpalis* and *G. morsitans*, respectively. *G. palpalis* prefers areas of vegetation near rivers and cultivated fields, and *G. morsitans* feeds on wild animals in savannah areas, far from human settlements.

Trypanosomiasis rhodesiense is a zoonosis, and humans visiting affected areas (usually for hunting or tourism) are accidental hosts. Humans are the only meaningful reservoir of *T. b. gambiense*. Untreated infections may persist for years. This disease is highly prevalent in Africa; ≈500,000 people are infected in 36 countries because of poor health systems in regions of civil and military turmoil (*1*). Despite its sporadic occurrence among travelers, *T. b. rhodesiense* has been reported more often in European (*2*) and American tourists (*3*) than *T. b. gambiense* because *T. b. rhodesiense* is present in areas not visited by expatriates. We report 2 cases of imported trypanosomiasis gambiense in Italy during the summer of 2004.

## Patient 1

On July 2004, a previously healthy 44-year-old man who lived in Gabon was admitted to the outpatient clinic of the university hospital in Udine, Italy, with a 6-month history of recurrent fever, headache, fatigue, weight loss, leg paresthesias, gait difficulties, and daytime somnolence. He had been living in Libreville, Gabon, since 1961, made yearly visits to Italy, and had never visited other African countries. He reported frequent tsetse fly bites while sailing on the Como River or walking in the forests in Gabon. He recalled several febrile episodes that had been presumptively treated as malaria; the last occurred in February 2004. The fever pattern then changed and became recurrent. The patient also had headaches and cutaneous hyperesthesia in the lower extremities. He subsequently had bilateral peripheral edema of the leg and progressive weakness, reversal of his sleep pattern with daytime somnolence and insomnia at night, loss of appetite, and a marked weight loss (20 kg). One month before admission to the hospital, he was examined in an emergency room and by a general practitioner, but a diagnosis was not made. Laboratory findings at that time were an erythrocyte sedimentation rate (ESR) of 122 mm/h and hypergammaglobulinemia (4.5 g/dL).

**Figure 1:**
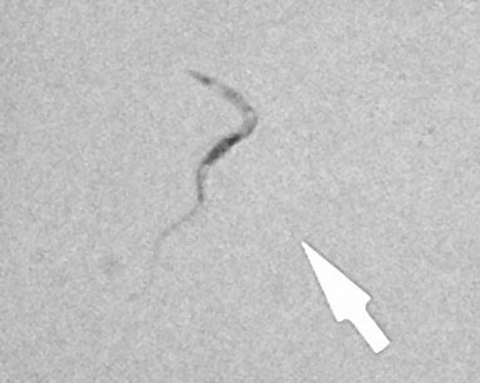
Trypomastigote (arrow) in a Giemsa-stained cerebrospinal fluid smear of patient 1 (original magnification ×1,000).

Upon examination, he was oriented but irritable and apyretic. He had a blood pressure of 110/70 mm Hg and a pulse rate of 104/min. Enlarged lymph nodes were found in the axillae, groin, supraclavicular region, and posterior neck triangle. The liver and spleen were enlarged (spleen diameter 20 cm by ultrasound). Neurologic examination showed walking ataxia, decreased sensitivity to light touch in both legs, and no deep tendon reflexes. Laboratory tests showed pancytopenia, an increased ESR, and hypergammaglobulinemia with increased levels of immunoglobulin M (IgM) (Table). Giemsa-stained blood films showed trypomastigotes. Lumbar puncture showed clear cerebrospinal fluid (CSF) with increased leukocyte counts, protein and IgM levels, and a low glucose level (Table). Trypanosomes were also found in the CSF (Figure 1). An indirect hemagglutination (IHA) test result was positive for *T. brucei* (titer 1:64). Second-stage sleeping sickness (stage 2 HAT) was diagnosed, but treatment with eflornithine (obtained from the World Health Organization [WHO]) could not be initiated until 9 days after the diagnosis because of getting medication through customs. In this 9-day period, daily peripheral blood smears were negative, except on day 5. The patient was then given a standard dose of eflornithine (100 mg/kg intravenously 4×/day for 14 days), and his condition improved rapidly, lymphadenopathy resolved, and neurologic status normalized within 2 weeks. Lumbar puncture on day 14 of treatment did not show any trypanosomes, and all CSF parameters improved. Repeat peripheral blood smears were also negative, and he was discharged. Two weeks later he was still healthy. He was advised to remain in Italy for further follow-up, but he went back to Gabon and has not provided any subsequent medical information.

**Table:** Laboratory test results for the 2 patients at admission*

Test (normal range)	Patient 1	Patient 2
Blood		
Leukocytes/μL (4.2–9.5 × 10^3^)	2.7	3.2
Hemoglobin, g/dL (13–17)	8.3	9.5
Platelets/μL (130–400 × 10^3^)	100	127
ESR, mm/h (<15)	131	46
CRP, mg/L (<5)	44.5	128
γ-globulins, g/dL (0.7–1.7)	3.8	1.5
Total protein, g/dL (6–8.5)	8.7	6.7
Total IgM, mg/dL (40–230)	1,161	681
Cerebrospinal fluid		
Protein, mg/dL (200–500)	839	330
Glucose, mg/dL (40–70)	49	68
Leukocytes/μL (0–2)	40 (90% lymphocytes)	0
IgM, mg/dL (0.5–1.5)	220	0.24
Trypanosomes	Present	Absent

*ESR, erythrocyte sedimentation rate; CRP, C-reactive protein; IgM, immunoglobulin M.

## Patient 2

A 54-year-old woman was admitted to the Centre for Tropical Diseases of Sacro Cuore Hospital of Negrar in Verona, Italy, in late September 2004 with a 3-month history of recurrent fever, headache, insomnia, and increased fatigue. She had lived for 30 years in the Central African Republic and had not visited any other African countries during that time. At admission, she was afebrile, and physical examination showed diffuse cutaneous hyperesthesia and splenomegaly (main spleen diameter 19.5 cm by ultrasound). Blood cell counts and biochemical tests showed anemia (hemoglobin level 8.3 g/dL) and leukopenia (leukocyte count 2,700/μL). A quantitative buffy coat test result for malaria was negative, and she was discharged.

**Figure 2:**
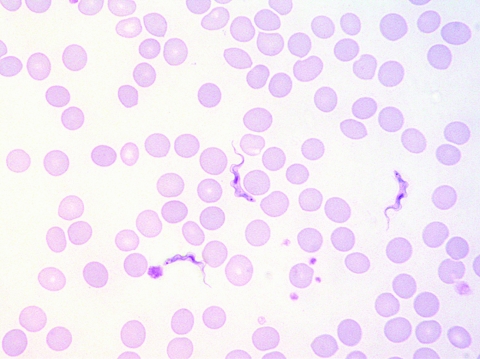
Trypomastigotes in a Giemsa-stained thin blood film of patient 2 (original magnification ×1,000).

Three days later she returned with a fever. A quantitative buffy coat test result was negative for malaria, but this test showed viable trypomastigotes. They were also found in peripheral blood smears (Figure 2). Serologic results for *T. brucei* (IHA test) were positive (titer 1:128). Other relevant laboratory findings are shown in the Table. Results of CSF examination were normal. Since we could not treat this patient with eflornithine (WHO provides this drug only for stage 2 HAT), intramuscular pentamidine was administered at the dose of 4 mg/kg for 10 days. Tests to detect trypanosomes in blood were conducted daily for 8 days after treatment was initiated, but no trypomastigotes were found. Her clinical course was uneventful, except for a sterile abscess at the injection site. All laboratory findings improved markedly. She was afebrile and was discharged on day 25 in good condition, although she still had insomnia and headaches.

In February 2005, she returned for a follow-up examination. The headaches and insomnia continued (she did not sleep >2 hours per night). Laboratory findings, including serum IgM levels, were within normal ranges. Total protein levels in CSF increased to 570 mg/dL, but cells in CSF were within normal ranges. Based on these findings, treatment with intravenous eflornithine (100 mg/kg 4×/day for 14 days) was initiated. The patient had generalized tremors (without fever) during the third infusion (no electroencephalographic signs of convulsions), but subsequent findings were uneventful. She was discharged after completion of treatment. At a follow-up visit in April 2005, she reported a nearly normal sleeping pattern.

## Conclusions

Eighty-four imported cases of trypanosomiasis caused by *T. b. gambiense* were reported in Europe before 1963. From 1966 to 1979, 12 cases were reported in France, which reported the most cases in Europe (*4*). During this period, incidence in trypanosomiasis-endemic countries decreased after intensive control activities. Eight imported cases of infection with *T. b. gambiense* in persons from Europe have been reported since 1985 (*4**–**11*), and 2 additional cases were recorded in France by the Centre National de Référence de l’Epidémiologie du Paludisme d’Importation et Autochtone (F. Legros, pers. comm.). To our knowledge, *T. b. gambiense* infection has not been reported in Italy (C. Mauro, Ministry of Health, pers. comm.).

Both patients denied visiting African countries where *T. b. rhodesiense* was present. A sporadic case of infection with *T. b. gambiense* in an Italian expatriate in Zaire (now the Democratic Republic of Congo) was reported in Belgium in 1996 (*12*). The simultaneous occurrence of 2 cases in Italy suggests an increased risk for infection with *T. b. gambiense* in expatriates working in disease-endemic areas. This increased risk is not surprising if one considers the increased incidence of this infection in African countries (*1*).

In countries not endemic for this infection, diagnosis of imported cases of infection with *T. b. gambiense* is challenging because of variations in clinical signs and symptoms and low sensitivities of diagnostic tests (*13*). The first patient in our study was misdiagnosed and not treated for several weeks because he had no fever and clinical manifestations were limited to neurologic symptoms. The second patient was also initially misdiagnosed. High levels of IgM in blood and an enlarged spleen should suggest the possibility of trypanosomiasis (the main differential diagnosis is hyperreactive malarial splenomegaly). Techniques for concentrating parasites should be used, and blood films should be examined daily in patients with these symptoms.

Involvement of the central nervous system in trypanosomiasis has been confirmed by increased lymphocyte counts (>5 cells/μL) or trypanosomes in CSF (*14*). However, as in the second patient, neurologic involvement cannot be ruled out even in those in whom CSF is normal. Better indicators of infection in blood and CSF are needed. Leukocyte counts >20 cells/μL in CSF and intrathecal IgM synthesis independent of trypanosomes in CSF have been proposed as modified criteria for diagnosis of stage 2 HAT (*14**,**15*).

Eflornithine, the preferred treatment for stage 2 HAT, was obtained from WHO to treat 1 of the patients. However, custom formalities in Italy, which resulted in a delay in receiving this drug, are inappropriate for emergency drug treatment. Thus, our experience with the 2 patients and recent outbreaks of infection with *T. b. rhodesiense* emphasize the need for readily available trypanocidal drugs.
